# The early conversion of deep-sea wood falls into chemosynthetic hotspots revealed by *in situ* monitoring

**DOI:** 10.1038/s41598-017-17463-2

**Published:** 2018-01-17

**Authors:** D. Kalenitchenko, E. Péru, L. Contreira Pereira, C. Petetin, P. E. Galand, N. Le Bris

**Affiliations:** 1 Sorbonne Universités, UPMC Univ. Paris 6, CNRS, Laboratoire d’Ecogéochimie des Environnements Benthiques, Observatoire Océanologique, 66650 Banyuls-sur-Mer, France; 2 Sorbonne Universités, UPMC Univ. Paris 6, 66650 Banyuls-sur-Mer, France; 3 Present Address: Université Laval, Département de Biologie, Québec, Canada; 4 Present Address: Laboratório de Hidroquímica-IO/FURG, Rio Grande, Brazil

## Abstract

Wood debris on the ocean floor harbor flourishing communities, which include invertebrate taxa thriving in sulfide-rich habitats belonging to hydrothermal vent and methane seep deep-sea lineages. The formation of sulfidic niches from digested wood material produced by woodborers has been known for a long time, but the temporal dynamics and sulfide ranges encountered on wood falls remains unknown. Here, we show that wood falls are converted into sulfidic hotpots, before the colonization by *xylophagaid* bivalves. Less than a month after immersion at a depth of 520 m in oxygenated seawater the sulfide concentration increased to millimolar levels inside immersed logs. From *in situ* experiments combining high-frequency chemical and video monitoring, we document the rapid development of a microbial sulfur biofilm at the surface of wood. These findings highlight the fact that sulfide is initially produced from the labile components of wood and supports chemosynthesis as an early pathway of energy transfer to deep-sea wood colonists, as suggested by recent aquarium studies. The study furthermore reveals that woodborers promote sulfide-oxidation at the periphery of their burrows, thus, not only facilitating the development of sulfidic zones in the surrounding of degraded wood falls, but also governing sulfur-cycling within the wood matrix.

## Introduction

The deep ocean hosts a variety of energy-rich habitats. Deep-sea fauna display a wide range of adaptations that enable them to take advantage of patchy and ephemeral resources. Among these habitats, sinking massive organic falls sustain diverse specialized invertebrates. Some of these specialized taxa have the capacity to degrade recalcitrant materials while others are members of the chemosynthetic lineages that dominate hydrothermal vent and methane seep communities^1–4^. In particular, wood debris were identified as a stepping stones in the colonization of the deep sea by invertebrates hosting sulfide-oxidizing symbioses. However, the temporal sequence and interplay of wood fauna with microbes driving sulfur-redox cycling remain largely unknown in deep-sea natural environments, preventing full accounting of the role of thiotrophy in energy transfer from wood to benthic communities.

High concentrations of reduced sulfur in wood matrices was first described from shipwrecks preserved in anoxic sediments over centuries^5,6^. Microbial diversity studies have also documented sulfate-reducing microbes inside wood logs experimentally deployed for 7 to 12 months at great depth under oxic conditions^7–9^. In this case, digestion of the ligno-cellulosic matrix by wood-boring bivalves^2,3,10^ that provide labile organic substrates to microbial degraders was considered a prerequisite for sulfide production^7,10^. The colonization by deep-sea *xylophagaid* bivalve starts within less than 2 months^11^, progressively leading to the accumulation of digested material inside their burrows and in the surrounding of wood logs, favoring the development of sulfidic conditions.

Recently, aquarium studies revealed an overlooked rapid production of sulfide from freshly-cut wood immersed in the absence of wood-borers^12,13^. Within a month in the microcosms, sulfide concentrations inside wood logs reach the millimolar range, similar to those occurring in other chemosynthetic fauna habitats, such as hydrothermal vents and cold seeps^12^. The development of sulfide-oxidizing biofilms at the wood surface within 30 days further supported the capacity of the labile components of wood to sustain chemolithotrophy^13^.

Despite the potential importance of this initial sequence, the early conversion of wood into a sulfide-rich habitat has never been explored *in situ* because of practical constraints in the implementation and monitoring of such fast colonization experiments at great depths. Previous experimental studies quantified sulfide from snapshot measurements after one year of immersion^7,14^; using this approach the authors were unable to document the complete temporal sequence

In this study we overcome this constraint by combining sulfide monitoring and time-lapse observation of wood log surface immersed at a depth of 520 m, at a rate of 2 to 4 times per day over 3 months, using autonomous sulfide sensors and a specially designed LED-camera device. The results shed light on the interplay of pioneer wood-boring colonists and the sulfur-cycling dynamics in natural conditions, at scales that have previously remained inaccessible.

## Results

### Rapid increase of sulfide inside wood after immersion

**Figure 1:**
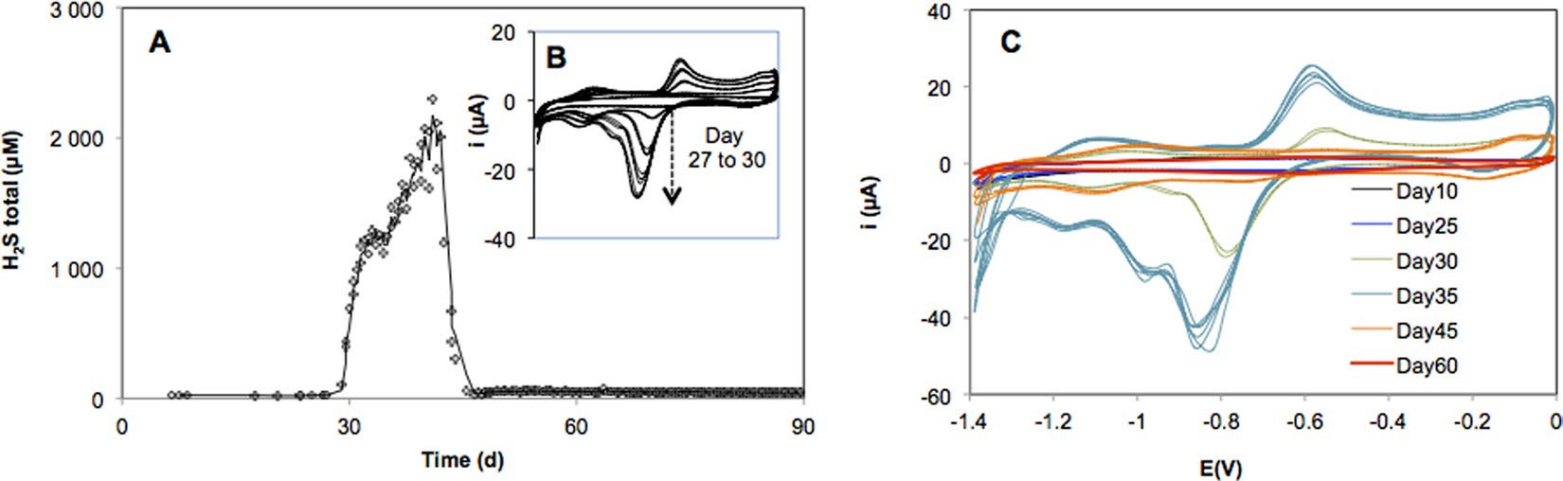
Evolution of the sulfide concentration inside the wood immersed at a 518 m-depth. The sulfide concentration increased rapidly from day 28 to day 41 followed by a steep return to background after 45 days (**A**). Consecutive voltammograms recorded over 3 days at the onset of the sulfidic period (**B**). Voltammograms recorded by the voltammetric electrode show the evolution of characteristic cathodic and anodic sulfide peaks over the 3-month experiment (**C**).

To track whether sulfidic conditions are established *in situ* within the timescale of microcosm experiments, we conducted autonomous voltammetric measurements on experimental wood logs deployed at a depth of 520 m in a Mediterranean submarine canyon. The Fig. 1 presents the continuous data series showing the development of sulfidic conditions inside wood over 3 months in these oxygenated natural conditions. Using a 0.8 mm diameter silver-disk electrode inserted 2 cm-deep into the wood log, we detected a steep increase of sulfide after approximately one month of immersion (Fig. 1A). The recorded cyclic voltammograms (i.e. the current-voltage curves obtained by cycling the electrode potential from 0 to −1.4 V) revealed the presence of negative (cathodic) and positive (anodic) current peaks characterizing free sulphide (H_2_S and HS^−^) (Fig. 1B,C). Starting from day 27, the voltammograms depicted the rapid rise of cathodic and anodic current peaks that denoted the increase of sulfide at the electrode (Fig. 1B). The corresponding sulfide concentration increased from undetectable to a maximum of ca. 2 mM within only 4 days, before rapidly returning to non-sulfidic conditions after 42 days (Fig. 1A,C).

### Microbial biofilm development in relation to surface chemical conditions

**Figure 2:**
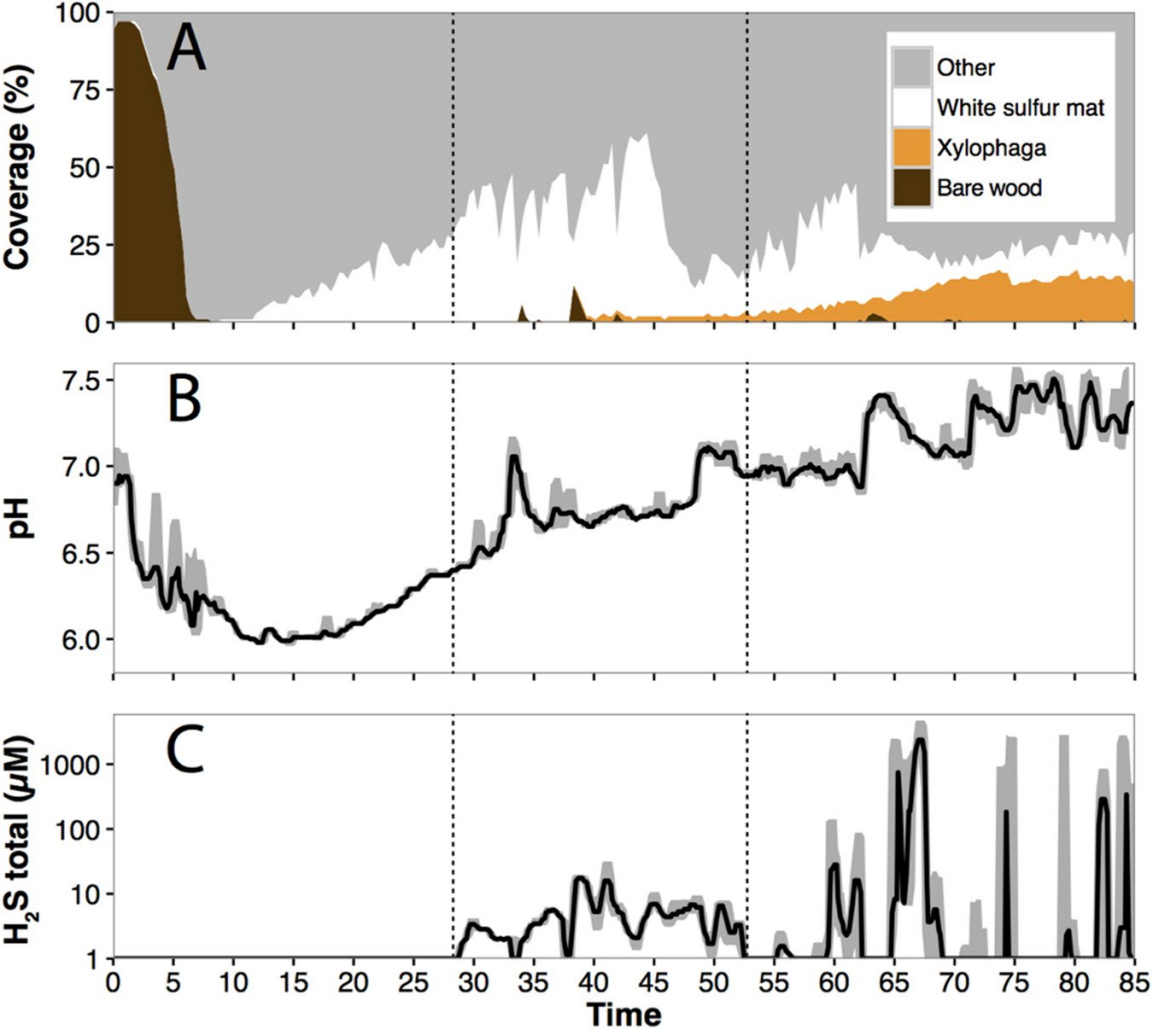
Observed changes at the surface of the experimentally immersed wood log over 85 days. (**A**) Percentage of bare wood (brown), white biofilm (white) and digested wood (orange). (**B**) pH and (**C**) sulfide concentrations recorded *in situ* at the surface of the log.

**Figure 3:**
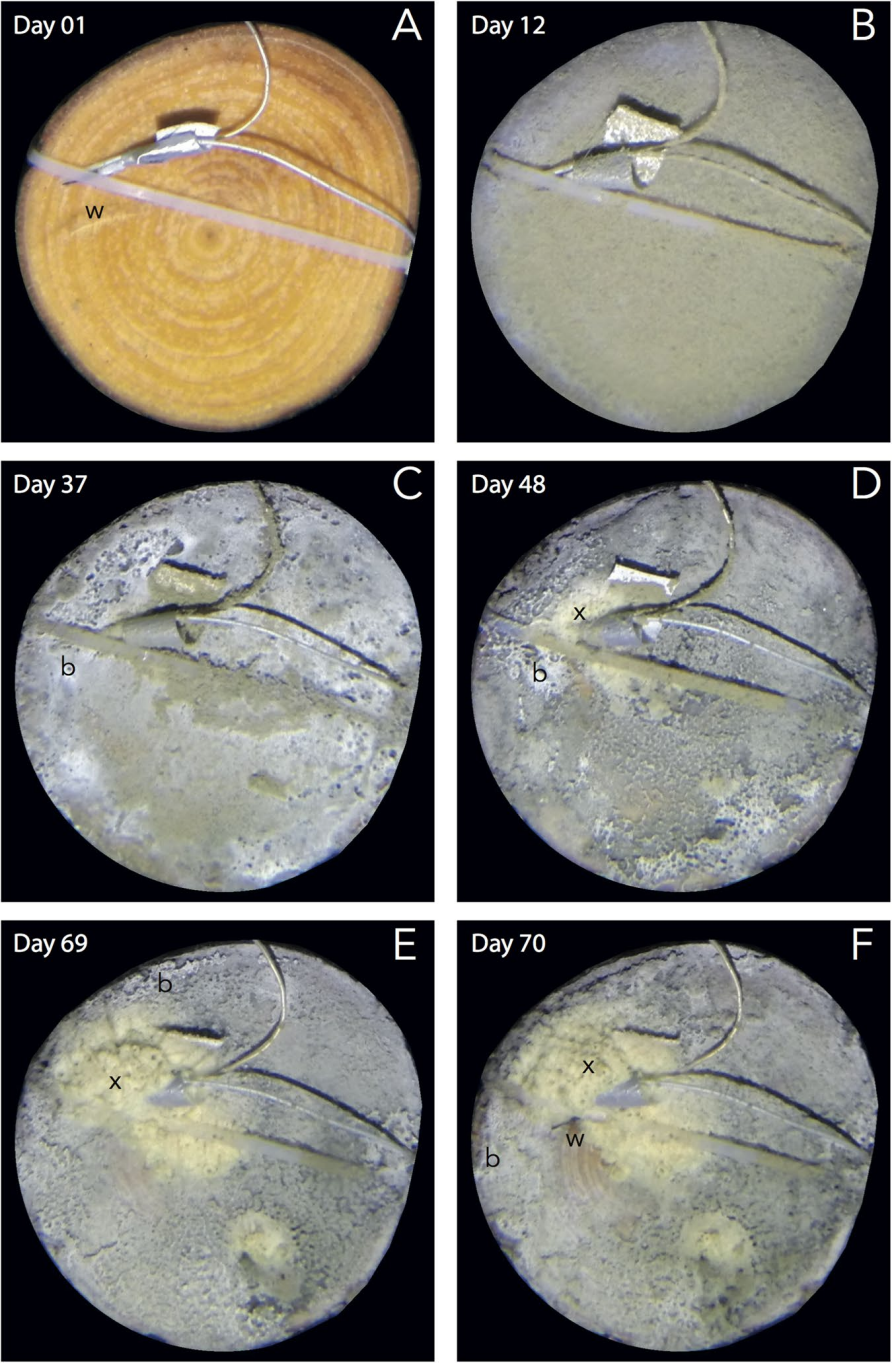
Images from the time-lapse series showing the development of the sulfur biofilm (b) (**A** to **C**) and the accumulation of digested wood material (x) around burrow orifices (**D** to **F**). On day 70, the electrode previously buried in digested wood material was re-exposed on bare wood (w) after a period of strong water flow (**F**).

To further investigate sulfide diffusion at the wood-water interface and sulfide use by chemolithotrophs under *in situ* conditions, we monitored the development of a microbial biofilm in combination with chemical changes on the wood surface. Our second experiment combined time-lapse imaging with sulfide and pH monitoring on the wood log over 3 months and was deployed at the same site as the first experiment (Supplementary Fig. S1, Supplementary Video S2). A white biofilm indicative of elemental sulfur deposition was observed after 11 days (Figs 2A and 3A,C) following a steep pH decrease to 6.0 (Fig. 2B). As the biofilm coverage expanded, the surface pH returned to neutral conditions. On the surface of the wood, sulfide was detected after 28 days (Fig. 2C). Up to day 52, the surface concentration remained in the micromolar range, three orders of magnitude lower than that measured inside the wood in the first experiment (Fig. 2C). In combination with the early deposition of elemental sulfur and pH decrease, low to undetectable sulfide concentrations revealed rapid sulfide redox-turnover at the wood-water interface.

**Figure 4:**
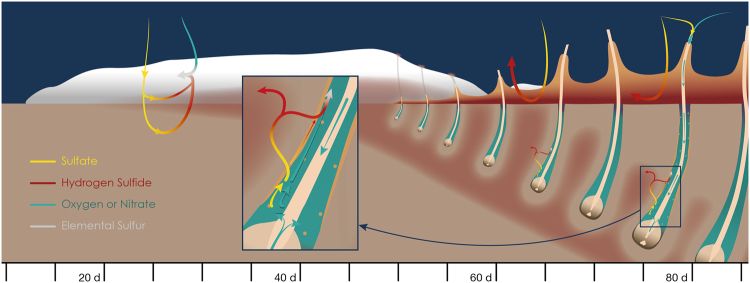
Successive stages driving the transfer of energy from freshly immersed wood to deep-sea biota over a 3-month period. The initial 1.5-month stage is characterized by the anaerobic degradation of labile components of wood, resulting in sulfide diffusion at the wood-water interface and its use by chemolithotrophic microbes forming a sulfur biofilm. In the second stage, wood-borers control the fluxes of electron acceptors through their burrows and drive the sulfide boundary deeper inside the wood. The wood-boring activity results in both the depletion of sulfide from the wood layer on the surface and the formation of a highly sulfidic layer of digested wood material on the log surface.

The establishment of the sulfur biofilm was even faster *in situ* than described in previous aquarium studies, which documented elemental sulfur on the surface of wood after 20 days^9^ (Fig. 4). Furthermore, videos and still images showed that the biofilm reestablished within 3 days after a strong hydrodynamic disturbance had swept it almost completely from the surface (Fig. 2 after days 34 and 39, Supplementary Video S2). Blackened patches, similar to the iron sulfide deposits observed on whale bones^15^, were also noticed on the wood surface after the biofilm was removed (Supplementary Video S2).

### Wood-borer influence on redox conditions at the wood-water interface

To unravel the role of wood-borers in sulfur redox-cycling during the early stages of colonization, we monitored the accumulation of digested wood material on the surface using the time-lapse image series recorded over 3 months. Detectable traces of wood-boring activity were first observed after 40 days (Fig. 3E,F, Supplementary Video S2), more than 4 weeks after the establishment of the biofilm.

We observed that the concentration of sulfide on the surface evolved to a more intermittent regime as the boring activity developed (Fig. 2C). The concentration of sulfide on the surface became much more variable, rapidly switching from non-sulfidic to millimolar sulfide levels. Examination of the time-lapse image series revealed that sulfide spiked to the millimolar range when the digested wood layer was covering the electrode (Fig. 3D) and sharply decreased when this layer was swept out from the electrode tip by strong water flows (Fig. 3E). The area surrounding the bivalve burrows appeared devoid of sulfide, in contrast to sulfide levels measured before colonization, which had been in the low micromolar range.

Additional voltammetric measurements on a wood log recovered after 85 day-deployment and placed in a 13 °C seawater aquarium confirmed that sulfide ranged from undetectable (i.e. <0.01 mM) to 0.1 mM inside live *Xyplophaga* burrows while sulfide reached 0.8 to 1.0 mM between the wood and bark in the uncolonized areas of the wood surface covered by the sulfur biofilm (Supplementary Table S4).

## Discussion

This study showed that sulfidic conditions suitable for sulfide-oxidizing microbes were established from the very early stages of wood immersion. The sulfide increase to millimolar levels within wood did not require the conversion of the ligno-cellulosic matrix into more labile digested organic material. This initial step of sulfide production is expected to be sustained by sulfate reducers fueled by the microbial consortia degrading the labile components of freshly-cut wood, like sugars, as described in aquarium experiments^9^, and within wood logs protected from borer colonization^16^.

Subsequently, the formation of a sulfur microbial biofilm reflected the use of sulfide at the wood surface by sulfide-oxidizers. The rapid regrowth of the biofilm after hydrodynamic disturbance indicated this chemolithotrophic activity is sustained over at least 1.5 months. Blackened patches similar to those observed on whale bones^15^ were visible below the biofilm detached from wood (Supplementary Video S2). These patches are typical of iron sulfide formation around sulfidic microniches in sediments^17^. Here, FeS was likely formed by the reaction of free sulfide with iron oxides coming from the thin sediment layer deposited at the wood surface. Unlike sediments, wood is largely devoid of iron^18^ and only a limited amount of iron sulfide was formed, most of the free sulfide remaining available for chemolithotrophic microbes. The biofilm thus constitutes the primary pool of labile organic matter available for wood colonizers (i.e. grazers and bacterivores as shown at methane seeps^19^), as illustrated by shrimps grazing on the biofilm occasionally detected on video sequences (Supplementary Video S6).

Our *in situ* experiments furthermore demonstrated that *Xylophaga* spp. colonization promoted the sulfide-oxidizing activity inside wood. In the presence of woodborers, sulfate reducers were previously shown to develop deeper inside the wood matrix^16^. Here we show that the sulfide produced in wood is not accumulating inside burrows. Instead, a depletion of sulfide from the wood surface is observed in the proximity of burrow orifices. Measurements performed on a wood log transferred in aquarium confirmed the low concentrations of sulfide inside the burrows, in comparison to the millimolar concentrations measured in the white biofilm area devoid of woodborers. These results suggested a deepening of the sulfur-redox horizon as the colonization by woodborers progressed, consistently with the steep decrease of sulfide observed 2 cm inside wood after 40 days in our first experiment (Fig. 4). We attributed this process to the advection of seawater electron acceptors via the external inhalant and internal exhalant siphons of *Xylophaga dorsalis*^20–22^ promoting sulfate reduction and sulfide oxidation using oxygen or nitrate at the periphery of burrows within the wood matrix. The yellow halos in areas previously covered by digested wood, which suggest the oxidation of iron sulfide precipitates around the burrow orifices, additionally supported this conclusion (Supplementary Fig. S3).

The role of *Xylophaga* sp. as ecosystem engineers is, therefore, more complex than considered^7,9,23^. In addition to the digested wood that favors the production of sulfide on the surface and around wood falls, deep-sea wood-borers sustain sulfur-cycling within the ligno-cellulosic matrix by driving the flow of electron acceptors deeper inside wood. As described from burrowing invertebrates in sulfidic sediments^24^, this process highlights the tight relationships between chemolithotrophic sulfide-oxidizing microbes and wood-boring species. It provides new clues to the habitat conditions that supported thiotrophic fauna evolutionary pathways, particularly for taxa living in sulfidic sediments such as the recently discovered giant *Teredinidae* shipworm hosting thiotrophic symbionts^25^.

These findings allow further comparison of wood fall habitats with other chemosynthetic habitats, and particularly whale falls, complementing primary assessments focused on the surrounding sediment^14^. The sulfide concentration inside pine wood reached several millimoles per liter after one month, a value similar to sulfide concentrations in sediments at a 4-year carcass (1 to 4 mM)^15^, and in the digested wood layer surrounding a 1-year massive experimental wood fall (0.8 to 5 mM)^7^. These sulfidic conditions, however, denote the microbial processing of degradation products dispersed over the surrounding sediments, 4 years and 1 year after the whale and wood falls reached the seafloor, respectively. A more relevant comparison of hard-substrate habitat conditions at the surface of wood is provided by whale bones, whose surface in the ‘sulfophilic’ stage harbors sulfur-rich bacterial mats. The concentration of sulfide was not measured inside bones or at their surface, but sulfate reduction fuelled by the degradation of lipids was confirmed in the first centimeter below the bone surface where seawater sulfate is available. However, this process is occurring when soft tissues have been completely consumed from whale carcasses, typically after several years^26^, while sulfidic conditions on wood falls developed within less than a month. Wood substrates further distinguish from whale bones by the rapid depletion of sulfide from the surface, even though it continues to be produced deeper in wood, resulting of the activity of *Xylophaga* spp. Among deep-sea hard substrate habitats sustaining sulfide-oxidizing bacterial biofilms, the surface of wood falls apparently share more similarities with the fresh basalts exposed to diffuse hydrothermal flows which similarly display micromolar sulfide conditions at temperature a few degrees above ambient and typically last a few months to 1 years after a volcanic eruption^27,28^.

Because future climate scenarios predict higher intensities for extreme meteorological events^29,30^, the ecological impact of fresh lignified material transported at depth, particular across narrow island shelves or through submarine canyons^31,32^, deserves increasing attention. Submarine canyon topography and hydrodynamic conditions are known to favor the export of organic material beyond the shelf to great depths^31^, and the transport of coarse fresh wood debris may be especially favored in areas where storm, hurricanes and flood events generate consequent land-to-sea export of these materials. Larvae pools of deep-sea wood boring bivalves were considered to be more abundant in these areas as regard to similar depths on the slope^33^. Conversely, the bacterial process that drive sulfide production from wood is not expected to be specific to local canyon conditions but might be ubiquitous in bathyal Mediterranean deep-sea conditions, as suggested by the occurrence of OTUs sequences related of sulfate-reducing bacteria *Desulfovibrio piezophilu*s in experimental wood logs deployed in the Lacaze Duthiers canyon experimental conditions^16^, in aquarium supplied with coastal waters^13^ and in natural wood falls in the deep-sea Nile fan at 1693 m depth^34^.

Overall, this study suggested a faster chemosynthetic energy transfer from wood debris to deep-seabed ecosystems than previously considered for organic falls. Futures studies should reconsider the importance of this initial chemosynthetic resource pulse in the first weeks of immersion of lignified plant debris as well as the role played by woodborers in the provision of chemosynthetic resource for wood colonizers and, more generally, for species sharing chemosynthetic habitats on the deep-seabed.

## Methods

### Wood immersion experiments

The study site is located approximately 13 miles offshore at the southern end of the Gulf of Lion in the western Mediterranean basin. In this area, submarine canyons incise the narrow shelf and establish tight connections between the coastal and bathyal ecosystems from 200 to 1000 m depths^35^ (Supplementary Fig. S1). Two pine wood (*Pinus pinea*) experimental deployments were conducted. For the two successive experiments, wood logs were placed on experimental frames deployed using the trawl of the M/V Minibex (COMEX) and positioned with the help of a SuperAchille ROV at two close locations in the Lacaze-Duthiers canyon head at depths of 518 m (42°32.723 N, 3°25.265 E) and 524 m (42°32.717 N, 3°25.306 E). At these depths, hydrological conditions correspond mostly to Western Mediterranean intermediate water conditions of the Western Mediterranean Sea (i.e. 13 °C, salinity 38.5. 195–215 µM oxygen), except during dense shelf-water cascading events, which did not occur during the duration of the experiments. Logs of similar size (i.e., 15 cm long by 10 cm diameter) were cut from live trees and immersed within 3 days based on the method described in Kalenitchenko *et al*.^22^. Logs fixed on the instrumented platforms lied 50 cm to 1 m above the sediment and were pictured with the ROV after deployment (Supplementary Fig. S1).

To document sulfide inside *Xylophaga* spp. burrows, we additionally used one of the ‘control’ wood logs of a borer-exclusion experiment that followed the same protocol at the same experiment site^16^. The wood log colonized by live woodborers after 85 days was transferred in a seawater aquarium at 13 °C and measurements were performed 12-day later.

### *In situ* sulfide monitoring

Total sulfide (i.e., HS^−^ + H_2_S) was monitored on immersed wood logs over the 3-month long experiments using autonomous underwater sensors (NKE S.A., Hennebont, France). We used voltammetric and potentiometric methods in the first and second experiments, respectively. For measurements inside the wood, we used cyclic voltammetry on a bare-silver disk, which is a pH-independent method adapted for the marine environment^36^. At the wood surface, we used a potentiometric Ag/Ag_2_S electrode with simultaneous pH measurement with a miniaturized glass electrode. Although less accurate than voltammetry, potentiometry has a better sensitivity to sulfide at micromolar concentrations. Both methods were previously demonstrated to be suitable for semi-quantitative assessments of the sulfide concentration under the experimental conditions^36,37^.

The voltammetric electrode was a 0.8 mm silver wire protected with heat-shrinkable Teflon connected to an underwater potentiostat (SPOT, NKE SA, France)^36^. The electrode tip was inserted 2 cm below the wood surface in a drilled hole adjusted to the outer diameter of the electrode. Triplicate voltammograms were obtained by sweeping the potential from −1.4 to 0 V after a cleaning step of 30 s at −1.2 V. For sulfide concentrations above 20–30 µM, voltammograms exhibit a characteristic cathodic peak at a potential ranging from −0.7 to −0.8 V, and above 1 mM, an anodic peak is additionally observed^36^. We recorded two triplicate voltammograms per day.

The same method was used for punctual measurements on a wood log maintained in aquaria. The silver electrode was inserted c.a. 1 cm inside a burrow and triplicate voltammograms were recorded every minute over 5 to 6 minutes. The same procedure was repeated for three burrows, one of them being located at the periphery of the colonized area close to the white biofilm (Supplementary Fig. S6). Two other measurement series were obtained after the electrode was inserted between the bark and the sapwood in the white biofilm area (Fig. S6).

The height of the cathodic sulfide peak was used to calculate the total concentration of sulfide. Sensor calibration was performed in the laboratory before deployment using standard additions of Na_2_S in 500 ml of filtered and deoxygenated seawater in a thermostated beaker maintained at the *in situ* temperature. The agitation was stopped during voltammogram acquisition to mimic the inner wood conditions. The stability of the electrode under the course of the experiment was estimated to be better than ±33%, based on the standard deviation of the slope determined from repeated calibrations of the same electrode over 3 years before and after deployment.

Potentiometric loggers (SPHT, NKE SA) recorded the potential of the sulfide or pH electrodes on the surface of wood at a rate of 4 measurements per day. Each logger was equipped with an Ag/AgCl reference electrode using seawater as an electrolyte. The pH and sulfide electrodes were combined and tightly attached at the surface of the log using a tie wrap (Supplementary Fig. S1). A pH glass electrode of 1.5 mm diameter (M1.5, INGOLD) was calibrated with standard additions of HCl (0.1 M) in seawater. The Ag/Ag_2_S electrode was a 0.8 mm-diameter silver wire (Goodfellow) protected with heat-shrinkable Teflon. The 5 mm-long unprotected tip was pre-conditioned overnight in a 200 mM Na_2_S solution^37^. The logarithmic Nerstian response of the sulfide electrode to the S^2−^ was calibrated at 13 °C in the laboratory before deployment using standard additions of Na_2_S in 500 ml of filtered and deoxygenated seawater, adjusting the pH to 7.0 ± 0.1 with HCl 0.1 M using an automated pH control system (Titrando, Metrohm). The calibration coefficients (slope and E°) and the *in situ* pH were used to convert the electrode potential to the total sulfide concentration^38^.

### Autonomous underwater camera

For the second experiment, we designed and built a dedicated autonomous monitoring system that integrated an underwater, miniaturized camera and an LED light, which equipped the platform. The camera was approximately 30 cm above the upper face of the logs. The autonomous camera (SPYDEEP) was composed of three modules (A, B and C) connected by a custom Y-cable equipped with Seacon (Westerly, RI, USA) 4 pin MCIL4FS connectors (Supplementary Fig. S7). Module A was composed of an extended version of the GoBenthic deep-sea enclosure (GroupBinc, Jensen Beach, FL, USA). A GoPro Hero 3+ (GoPro, San Mateo, CA, USA) was programed to acquire 3 pictures and a 20-second video every 9 hours. A custom script on the camera SD card was used to program the sequence, and a hardware time-lapse controller (Camdo, Vancouver, BC, Canada) plugged into the GoPro main connector controlled the camera wake-up every 9 hours. Module B included a lithium polymer battery (Hacker Motor, Ergolding, Germany) with four 3.7 V energetic cells of 5000 mAh mounted in parallel, which was encased in a GPH-1250 m underwater housing (Groupbinc, Jensen Beach, FL, USA). Module C included a Nano SeaLite lamp (Deepsea Power & Light, San Diego, CA, USA). The battery was connected with the Y-cable to the camera through a power adaptor (Camdo, Vancouver, BC, Canada) that converted the 14.8 V DC voltage of the battery into a regulated 4.3 V DC voltage. The lamp module was also connected to the battery through the converter. We used a MOSFET transistor (Sparkfun, Niwot, CO, USA) to close the circuit and turn the lamp on only when the camera was powered.

### Indicators of microbial and *Xylophaga* colonization

We defined four types of wood surface cover that could be quantified from underwater images. “Bare wood” indicated the wood surface devoid of any overlying material. “White sulfur mat” indicated the wood surface covered by a microbial biofilm, the white color being attributed to elemental sulfur and was quantified from the number of white pixels on the wood surface. “*Xylophaga*” represented the proportion of the surface covered by digested wood expelled from burrows. We measured the area corresponding to these cover types on one picture from each triplicate of the time-lapse using ImageJ software (V 1.50 g) (Supplementary Fig. S8).

The electrodes, tie wraps and tape were masked before the images were analyzed (Supplementary Fig. S8A). We used the color-threshold V1.6 plugin to set a user-defined color range discriminating the different cover types. Threshold values of the Hue, Saturation and Lightness parameters provided in Supplementary Table S10 were optimized for our experimental conditions (i.e. specific light intensity and position, camera distance from wood).

On every selected image, ImageJ calculated the area corresponding to the color thresholds using this plugin. “Other” defined the area that was not attributed to any of the three cover types and usually depicted wood covered by a thin sediment layer. Each image from the sequence was checked for assignment errors. We observed assignment errors only for digested wood. ImageJ erroneously attributed fresh sediments covers in the early stage of deployment to digested wood (Supplementary Fig S8D). The sulfur biofilm rapidly covered these sediments and no further error was identified after the first visual evidence of woodborer activity.

### Data availability

The datasets generated and analyzed during the current study are available from the corresponding author on reasonable request.

## Electronic supplementary material

Supplementary information

Supplementary Video S2

Supplementary Video S5

Supplementary Material
