# Supplementary material for: The early conversion of deep-sea wood falls into chemosynthetic hotspots revealed by in situ monitoring
Source: Sci Rep. 2018 Jan 17;8:907. doi: 10.1038/s41598-017-17463-2 (PMC5772046; doi:10.1038/s41598-017-17463-2)
Supplement: Supplementary file 1 — Supplementary information [file 41598_2017_17463_MOESM1_ESM.pdf]

The early conversion of deep-sea wood falls into chemosynthetic hotspots revealed by *in situ* monitoring

Authors: D. Kalenitchenko, E. Péru, L. Contreira Pereira, C. Petetin, P. E. Galand, N. Le Bris

Supplementary information

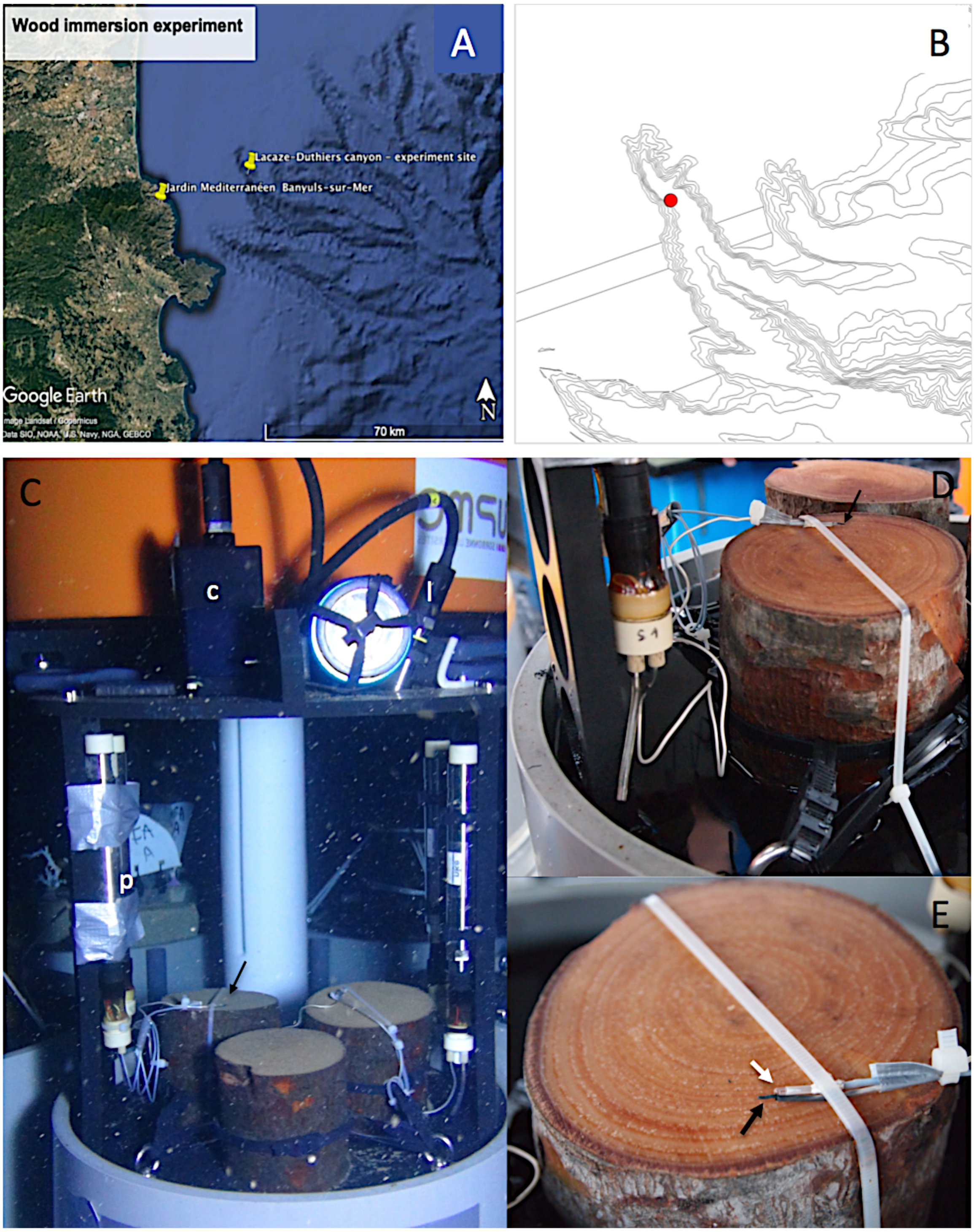

Supplementary Fig. S1. Location of the experiment site and pictures of the experimental set-up. A: map of the area in the Gulf of Lion showing the experiment site in the Lacaze-Duthiers canyon and the Banyuls-sur-Mer areas where the wood logs have been sampled, at the eastern end of the Pyrenean mountain (Adapted from Images ©2017 Landsat / Copernicus, Google, Data SIO, NOAA, U.S. Navy, NGA, GEBCO, Données cartographiques ©2017 Google, Inst. Geogr. Nacional). B: location of experiments inside the Lacaze-Duthiers canyon (Adapted from Berné, S. & Satra, C. Gulf of Lions bathymetry (GIS shape files) 2003). Setup of the second experiment at a 520 m-depth (C) and before deployment (D-E). The instrumented platform is holding the autonomous potentiometric sensors (p) and the camera and the LED light (c). The arrows indicate the tips of the 1.5-mm glass electrode (white arrow) and 0.8-mm sulfide electrodes (black arrows) on the surface of the monitored wood log (B-C).

Supplementary Video S2. Time-lapse of daily pictures over the 3-month experiment showing the evolution of the wood surface from day 1 to day 86.

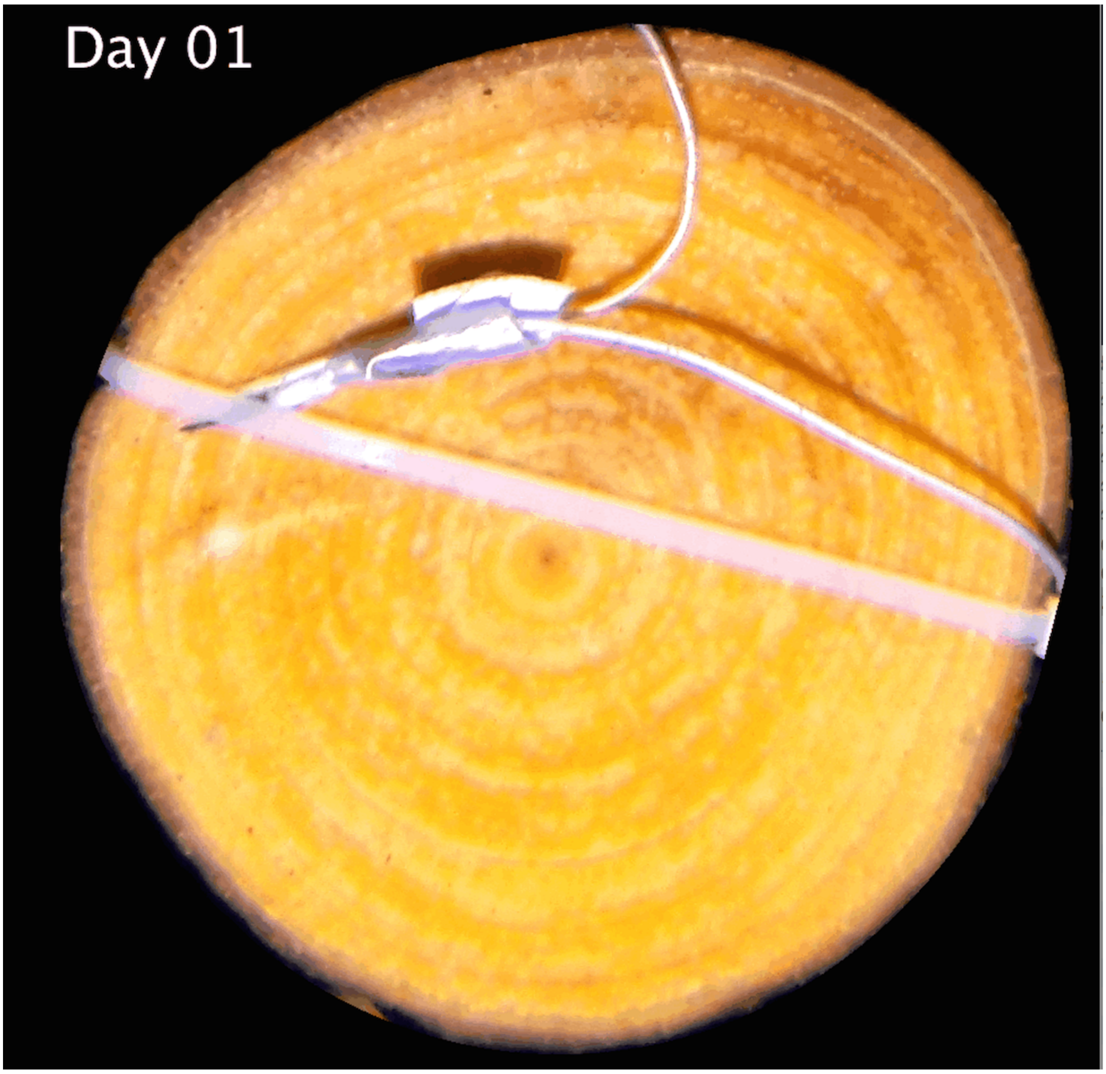

Supplementary Fig. S3. Surface of the wood log before (A) and after (B) the closure of the platform lid on day 85.

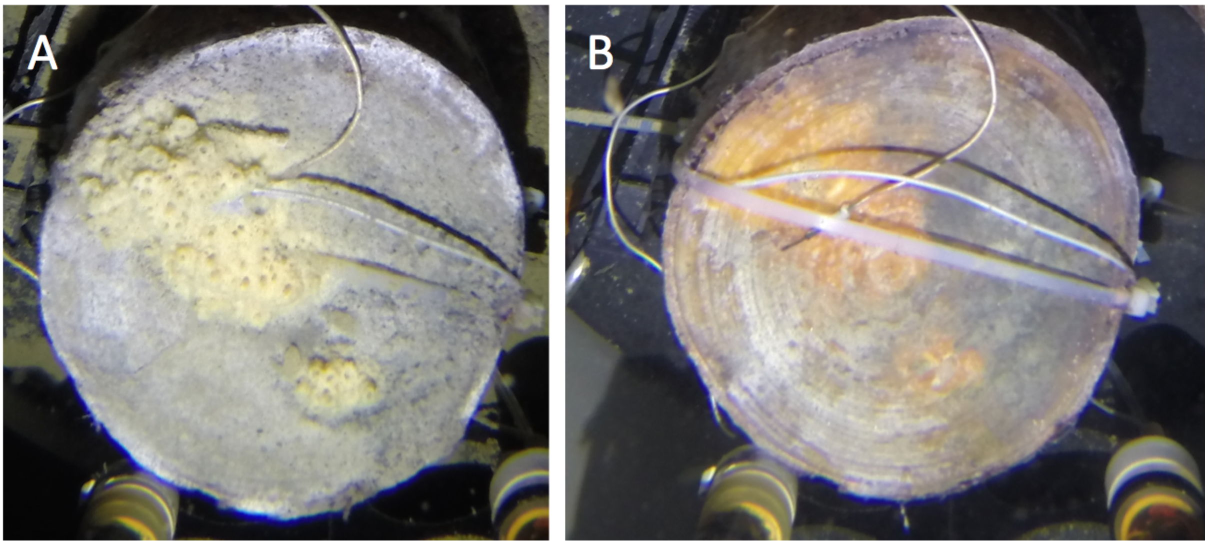

Supplementary Table S4. Sulfide measurements on the wood log transported in aquarium: S1, S2 and S5 inside *Xylophaga* spp. burrows, S3, S4 between the bark and sapwood in the sulfur biofilm zone devoid of burrows (Fig. S5).

Location	Sulfide (μM)<br>Mean +/- sd	n
S1	nd	47
S2	106 +/- 18	15
S3	822 +/- 85	15
S4	1024 +/- 75	12
S5	nd	20

Supplementary Video S5. 10s video record showing a shrimp grazing on the microbial biofilm.

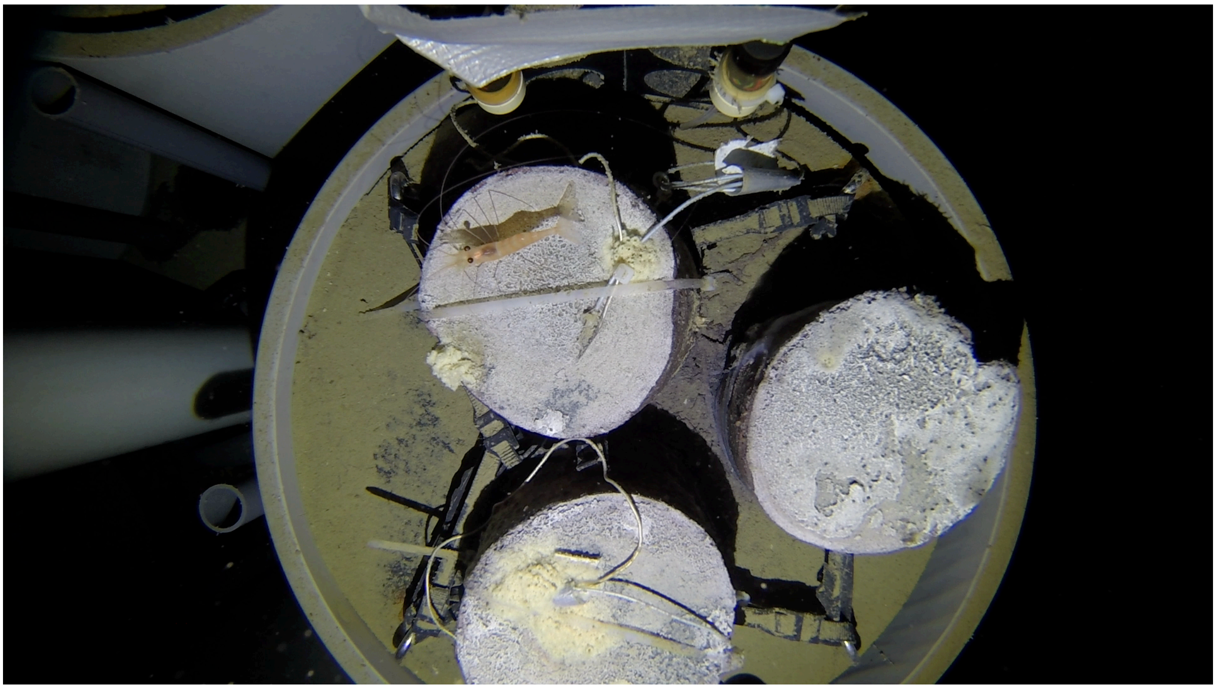

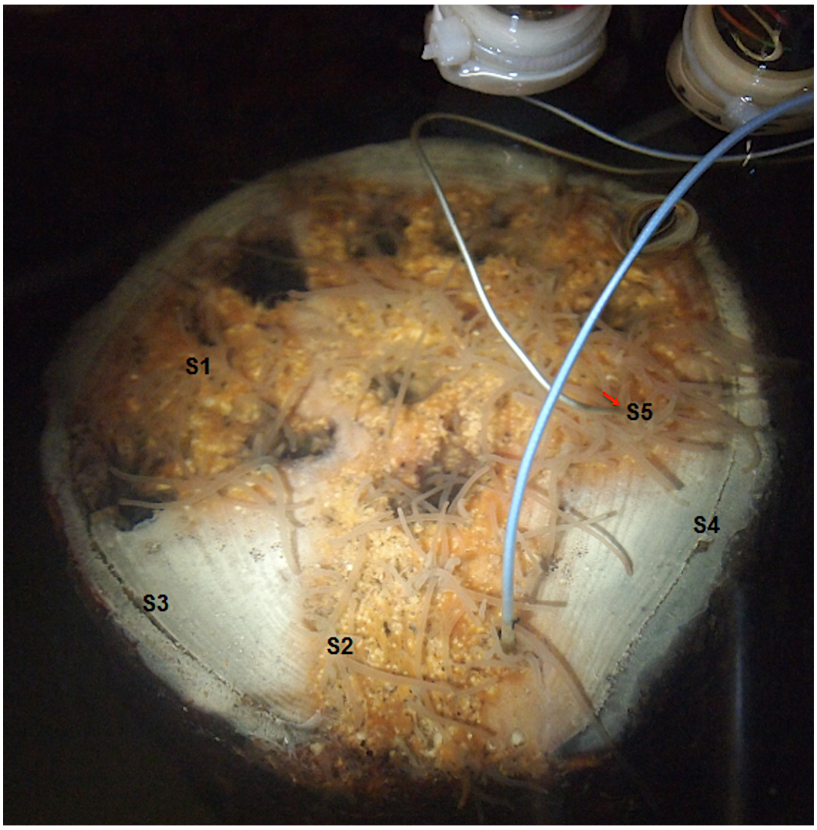

Supplementary Fig. S6. Location of sulfide measurements on the wood log transported in aquarium after a 85-day immersion at 520 m depth. The sulfide electrode (red arrow) was inserted 1-cm inside two burrows (S1 and S5) in the middle of the orange-yellow region of the log surface characterized by a high density of *Xylophaga* spp. siphons. One measurement was acquired in a third burrow located at the periphery of the white biofilm region devoid of woodborers (S2). S3 and S4 measurements were obtained between the bark and sapwood in the white biofilm region away from burrows.

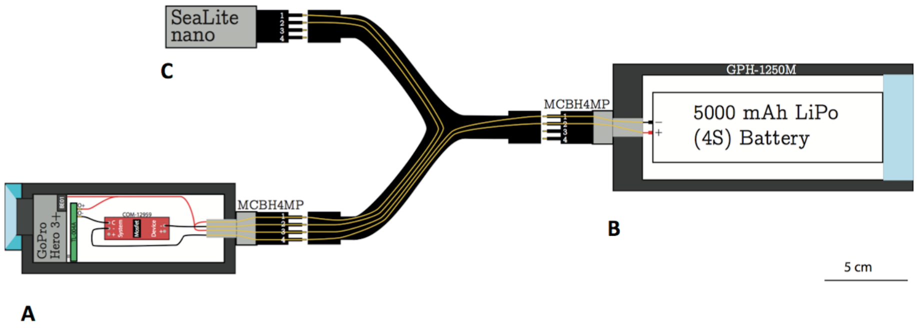

Supplementary Fig. S7. Wiring scheme of the autonomous camera, battery and LED light.

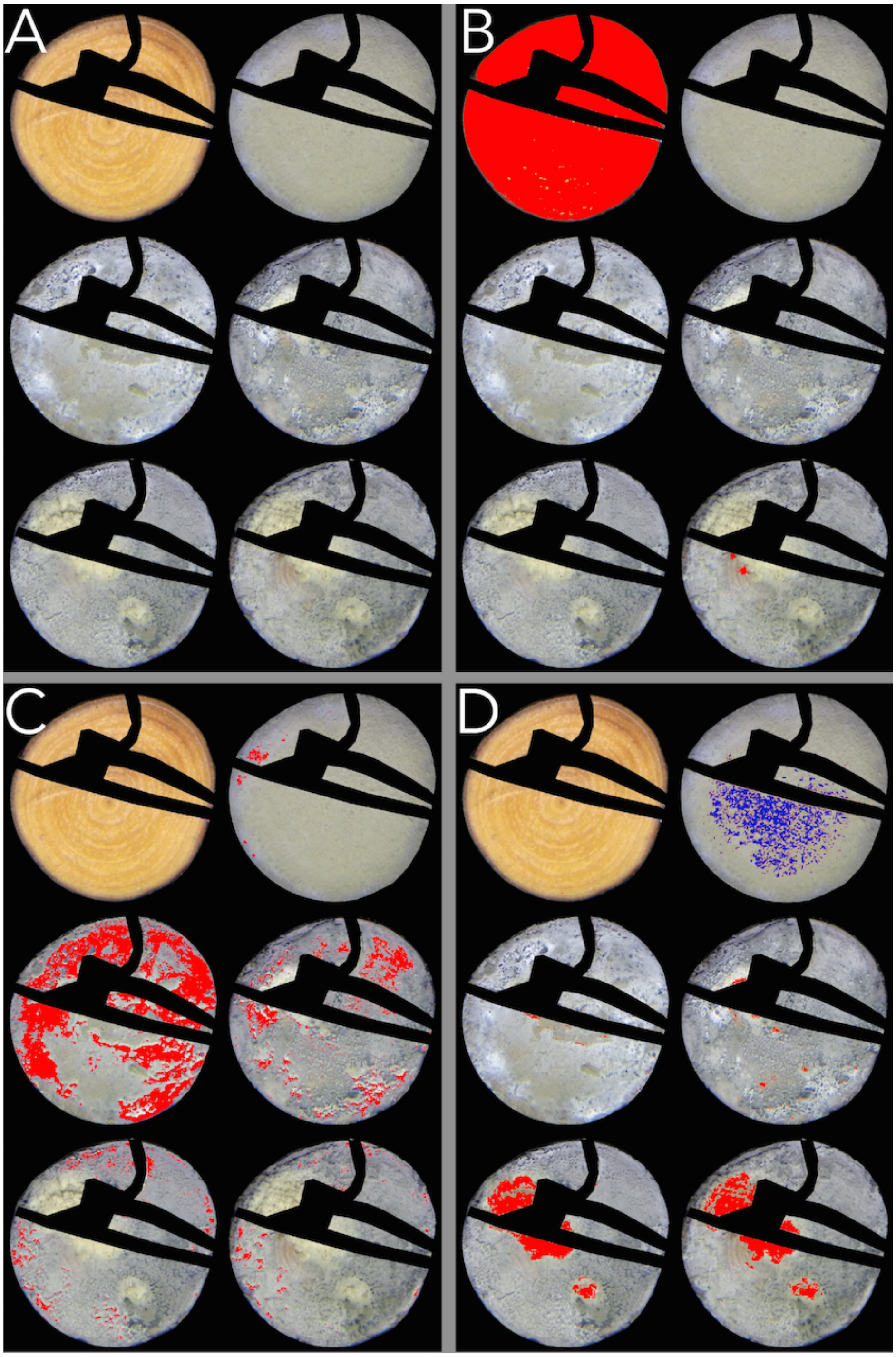

Supplementary Fig. S8. Quantification of the indicators of microbial and *Xylophaga* colonization defined with ImageJ. After a black neutral mask was applied to electrodes, tie wraps and tape on the image series (A), the area corresponding to the thee cover types '"Bare wood" (B), "White sulfur mat" (C) and "*Xylophaga* (D) were automatically mapped and quantified using optimized color thresholds. Sediment deposits lead to assignment errors at the beginning of the experiment (blue color in D). The quantification of digested wood after 40 days was not affected by such artifact (red color in D).

Supplementary Table S9. Threshold parameters used to define the different covering features.

Covering feature	Hue	Saturation	Lightness
Bare wood	0-29	63-255	108-255
White biofilm	141-255	0-255	185-255
Digested wood	31-46	25-245	180-255
